# CD4+ levels control the odds of induction of humoral immune responses to tracer doses of therapeutic antibodies

**DOI:** 10.1371/journal.pone.0187912

**Published:** 2017-11-09

**Authors:** Sharat Srinivasula, Erin Gabriel, Insook Kim, Paula DeGrange, Alexis St Claire, Candace Mallow, Robert E. Donahue, Chang Paik, H. C. Lane, Michele Di Mascio

**Affiliations:** 1 Clinical Research Directorate/Clinical Monitoring Research Program, Leidos Biomedical Research, Inc., NCI Campus at Frederick, Frederick, Maryland, United States of America; 2 Applied/Developmental Research Directorate, Leidos Biomedical Research, Inc., NCI Campus at Frederick, Frederick, Maryland, United States of America; 3 Integrated Research Facility, National Institute of Allergy and Infectious Diseases, National Institutes of Health, Frederick, Maryland, United States of America; 4 Division of Clinical Research, National Institute of Allergy and Infectious Diseases, National Institutes of Health, Bethesda, Maryland, United States of America; 5 Hematology Branch, National Heart Lung and Blood Institute, National Institutes of Health, Bethesda, Maryland, United States of America; 6 Radiopharmaceutical Laboratory, Nuclear Medicine, Radiology and Imaging Sciences, Clinical Center, National Institutes of Health, Bethesda, Maryland, United States of America; 7 Laboratory of Immunoregulation, National Institute of Allergy and Infectious Diseases, National Institutes of Health, Bethesda, Maryland, United States of America; 8 Biostatistics Research Branch, Division of Clinical Research, National Institute of Allergy and Infectious Diseases, National Institutes of Health, Rockville, Maryland, United States of America; Emory University School of Medicine, UNITED STATES

## Abstract

Rapidly increasing number of therapeutic antibodies are being repurposed to imaging probes for noninvasive diagnosis, as well as monitoring during treatment or disease recurrence. Though antibody-based imaging involves tracer doses (~3 log lower than therapeutic doses), and immune responses are severely reduced in patients with impaired immunity, formation of anti-tracer antibodies (ATA) has been observed hampering further diagnostic monitoring. Here, we explored the potential to develop humoral responses to intravenously administered tracer dose of a monoclonal antibody F(ab΄)_2_ fragment, and associated with host related immune measures in 49 rhesus macaques categorized into healthy (uninfected controls), SIV-progressors, SIV non-progressors, or total body irradiated (TBI). Antibody fragment administered in tracer amount (~100μg) induced immune responses with significantly lower odds in SIV-progressors or TBI macaques (P<0.005) as compared to healthy animals. Peripheral blood (PB) CD4+ cell counts, but not CD20+ cell levels, were associated with significantly higher risk of developing a humoral response (P<0.001). Doubling the PB CD4+ counts is associated with an odds ratio of developing an immune response of 1.73. Among SIV-infected animals, CD4+ cell count was a stronger predictor of immune response than plasma SIV-RNA levels. Both SIV-progressors and TBI macaques showed higher odds of responses with increasing CD4+ counts, however when compared to healthy or SIV non-progressors with similar CD4+ count, they were still functionally incompetent in generating a response (P<0.01). Moreover, presence of ATA in systemic circulation altered the *in vivo* biodistribution by increasing hepatic uptake and decreasing plasma radiotracer clearance, with minimal to no binding detected in targeted tissues.

## Introduction

The adaptive immune system of jawed vertebrates, indispensable for survial and though emerged long ago, has been remarkably uniform for millions of years[1]. The ability to provoke and direct a highly specific immune response against an antigen is vital, and is a core component of vaccine development. But such a response is undesirable and unsafe when the antigen is a therapeutic biologic. With rapidly increasing protein therapeutics among medicinal products[2], immune responses elicited towards the therapeutic may result in reduced efficacy, potency, and in rare cases, adverse effects of anaphylaxis and autoimmunity[3]. Even some FDA-approved biologics for use in the treatment of human disease, despite being humanized or fully human derived such as recombinant human cytokines or human antibodies, have been shown in clinical studies to be highly immunogenic in patients[4–6]. Immunogenicity of therapeutic proteins is characterized by the development of highly specific anti-therapeutic antibodies with a high affinity.

IgG class monoclonal antibodies (mAbs) are widely used from diagnostics to cancer treatment as they are highly specific to their targets and confer Fc-mediated effector functions[7–9]. Intact mAbs have long residence time ranging from a few days to weeks, and so are preferred for therapy. In contrast, antigen-binding fragments have much faster clearance in the blood, have reduced non-specific binding resulting in higher specific-to-nonspecific uptake ratios relatively soon after the injection, have lower risk of immunogenicity *in vivo*, and thus are preferred for diagnostic imaging. Since the pharmacokinetic and safety profile of FDA-approved therapeutic antibodies is already known, these therapeutic mAbs are being repurposed to imaging agents [10, 11]. Antibody-based radiotracer imaging provides a noninvasive diagnosis for establising the presence, location, molecular characterization and quantitization of cell surface phenotype *in vivo*, and these radiotracers are fundamentally different from therapeutic mAbs in that they are given infrequently, are administered at tracer dose (2–3 log lower than therapeutic dose), and are designed to measure molecular processes, not to modify or treat the disease.

Patients with impaired immunity, either due to immunosuppressive medication, chronic HIV infection, total body irradiation (TBI), or organ transplantation are at a greater risk of opportunistic infections with more severe consequences compared to healthy individuals. Though the impact of infections could be reduced by vaccination, the desired antibody response is severely reduced due to impaired immunity[12, 13]. Alternatively, formation of antibodies against the radiotracer (anti-tracer antibodies) limits further patient monitoring as they can lead to wrong diagnosis. Here, we explored the potential to develop immune responses to intravenously administered tracer dose of a monoclonal antibody F(ab΄)_2_ fragment (T-cell dependent antigen) directed against CD4 receptor, and associated with host related immune measures in 49 rhesus macaques grouped into healthy (uninfected controls), SIV-progressors, SIV non-progressors, or TBI. To accomplish this, two highly sensitive immunogenicity assays were developed.

## Methods

### Animals

Forty-nine Indian rhesus macaques (*Macaca mulatta*) were used in this study following both National Institute of Allergy and Infectious Diseases (NIAID), and National Heart, Lung, and Blood Institute (NHLBI) Animal Care and Use Committee approved protocols for *in vivo* SPECT imaging of whole-body CD4 pool using a F(ab΄)_2_ antibody fragment of either a depleting rhesus recombinant immunoglobulin G1 (rhIgG1) anti-CD4 (CD4R1), or a non-depleting humanized immunoglobulin G4 (huIgG4) anti-CD4 (huOKT4A) monoclonal antibody labelled with ^99m^Tc. At recruitment, thirty-two macaques were healthy uninfected controls and seventeen were infected with SIV/SHIV strains. Of the 32 healthy, 24 macaques (13 male, 11 female, age at first exposure = 2.8–18 years) remained healthy while 8 macaques (all male, age at first exposure = 2.7–5.1 years), after baseline imaging, underwent varying doses of total body irradiation (TBI) followed by autologous stem cell transplantation and post-transplant imaging. With one animal already developed antibody response to radiotracer after baseline imaging (but prior to irradiation) and was not followed longitudinally, and another macaque euthanized soon after post-transplant imaging, the remaining 6 TBI animals were followed longitudinally to evaluate noninvasively the CD4+ repopulation in lymphoid tissues. Of the 17 SIV/SHIV infected, 11 animals were infected with SIVmac251, 2 animals with SIV-TK, 1 animal with SIVmac239, 1 animal with SIVE543, 1 animal with SHIVAD8, and 1 animal co-infected with SHIVDH12R\SIVmac239. 5 SIV-infected macaques (all male, age at first exposure = 5.8–10.2 years) had nadir peripheral blood CD4+ count >600 cells/μl during the chronic phase of untreated infection and were termed non-progressors, while the remaining 12 SIV-infected macaques (5 male, 7 female, age at first exposure = 5.0–11.1years) had nadir CD4+ count <500 cells/μl during the chronic phase of untreated infection and were termed progressors. Nine of 12 SIV-progressors were imaged longitudinally to examine the dynamics of lymphoid tissue CD4+ pool following initiation or interruption of combination antiretroviral therapy (cART). Animals in the SIV-progressor and non-progressor groups are mutually exclusive, and can be either cART treated or untreated at the time of exposure or changing their therapy status in-between multiple exposures. Three additional rhesus macaques were also imaged using intact-huOKT4A labelled with ^111^In.

### Welfare of non-human primates

Animals were housed and cared for according to the *Guide for the Care and Use of Laboratory Animals*, 8^th^ Ed., Animal Welfare Act regulations, and NIH policies, an AAALAC accredited facility. All procedures were performed according to both NIAID and NHLBI Animal Care and Use Committee approved animal study protocols. Animals were anesthetized for all handling and manipulations, including imaging, and analgesia provided for any procedures that are considered to cause pain or distress to humans. Anesthesia was initiated with restraint dose of ketamine (10 mg/kg), and then maintained with propofol (0.2 mg/kg) via the catheter inserted in the saphenous vein of the leg. For humane, painless and rapid euthanasia, the sedated animal is injected with pentobarbital sodium 390mg/mL administered intravenously at a dose of 1mL per 5kg of body weight. Animals were housed in 6.0 sq ft stainless steel cages on a 12hr light/dark cycle and fed Purina Old World Primate Diet (Purina Mills, St. Louis, MO) with enrichment food (bananas, grapes, oranges, apples, peanuts, vegetables) and toys (hanging mirrors, kong toys, nylabones, puzzle balls, perches, and PVC toys) provided daily. Animals were pair housed whenever possible.

### Antibodies

To produce rhesus recombinant anti-CD4 (CD4R1), complementary determining regions (CDRs) of OKT4A, a murine anti-CD4 monoclonal antibody, were grafted onto rhesus IgG1 constant and variable framework. Humanized anti-CD4 (huOKT4A), where CDRs of murine OKT4A were grafted onto human IgG4 constant and variable regions was produced from transfected murine myeloma cell line. Hence both humanized and rhesus recombinant anti-CD4 antibodies have murine content, and bind to the same epitope on D1 domain of CD4 receptor. Preparation of F(ab΄)_2_ fragments by pepsin digestion, conjugation of HYNIC to F(ab΄)_2_, and ^99m^Tc labelling was previously described[14]. Of the 49 animals, 9 animals received F(ab΄)_2_-huOKT4A, 37 animals received F(ab΄)_2_-CD4R1, and remaining 3 animals received both F(ab΄)_2_ antibodies (during our transition from humanized anti-CD4 to rhesus recombinant anti-CD4) for a total of 118 exposures (28 exposures with F(ab΄)_2_-huOKT4A and 90 exposures with F(ab΄)_2_-CDR41). Preparation of ^111^In labelled, DTPA conjugated, intact-huOKT4A was previously described[15].

### Animal study design

^99m^Tc labelled antibody F(ab΄)_2_ fragment (radiotracer) was administered intravenously in the saphenous vein of the leg. Animals received radiotracer injection in the afternoon between 2–5 pm, and were imaged at 4h post-radiotracer injection. TBI studies were designed to longitudinally image CD4+ cell recovery following varying doses of total body irradiation/reinfusion of stem cells (CD34+) with a radiotracer administration pattern of baseline imaging followed by TBI/transplantation, and at ~week 1, months 1, 3, 5, 9 and 12 post-TBI[14]. In the SIV non-progressors, 4 of the 5 macaques were NOT on cART, whereas the remaining 1 macaque was on cART at the time of their exposure(s). In the SIV-progressor group, 3 of the 12 macaques were NOT on cART at the time of their only exposure, whereas the remaining 9 progressors were part of longitudinal study to image lymphoid tissue CD4+ pool recovery (or depletion) following initiation (or interruption) of cART with a radiotracer administration pattern of baseline exposure followed by cART start (or interruption), and month 1, 3, 5, 9, and 12 post-intervention. 7 of the 9 SIV-progressors in this longitudinal study initiated cART after their baseline exposure, whereas the remaining 2 progressors interrupted cART after their baseline scan. Some animals in this longitudinal study underwent multiple baseline scans (before therapy intervention). cART regimen was Tenofovir (PMPA) 20 mg/kg + Emtricitabine (FTC) 30 mg/kg subcutaneously once a day, and Raltegravir 20 mg/kg/BID mixed in food. Animals that underwent multiple exposures were tested before subsequent exposures for the presence of anti-tracer antibodies (ATA) from prior exposures. To check for changes in biodistribution, some animals were intentionally exposed and imaged while ATA from prior exposure were present in the blood. Since one animal already had an immune response before TBI, only 7 of the 8 total body irradiated animals started contributing to TBI group (and simultaneously stopped contributing to healthy group). The total 49 animals were divided into 4 groups–healthy (n = 32; 24 animals that remained healthy uninfected controls, plus 8 TBI animals until they underwent irradiation), TBI (n = 7), SIV-progressors (n = 12) and non-progressors (n = 5). To visualize the changes in *in vivo* biodistribution while ATA were present in plasma, three additional animals were imaged with ^111^In labelled intact-huOKT4A at 48h post-injection at baseline and 5 weeks later.

### Immunogenicity assays

#### Binding antibody assay (radio-HPLC)

To detect all anti-tracer antibodies that bind to the radiotracer, a size-exclusion high performance liquid chromatography with radioactivity detector assay was performed with plasma pre-incubated with the radiotracer. Aliquots of ^99m^Tc -F(ab΄)_2_-CD4R1 or ^99m^Tc -F(ab΄)_2_-huOKT4A were incubated with monkey plasma at a concentration of ~1.5nM for 30 min at 37°C in a humidified 5% CO_2_ incubator. A 50μl aliquot of radiotracer-plasma mixture was run through size-exclusion HPLC using a column (TSK gel G3000SW_XL_ column (7.8 x 300 mm, 5 μm, TOSOH Bioscience, Japan), 0.067 M sodium phosphate/0.15 M sodium chloride, pH 6.8; 1.0 ml/min) equipped with an on-line flow radioactivity detector (Bioscan, Inc. Washington DC).

#### Cell-based neutralizing antibody assay (plasma binding assay, PBA)

To detect anti-tracer antibodies that block tracer-target interactions, a cell-based neutralizing antibody assay was performed with plasma pre-incubated with the radiotracer. Aliquots of ^99m^Tc -F(ab΄)_2_-CD4R1 or ^99m^Tc -F(ab΄)_2_-huOKT4A were incubated with monkey plasma at a concentration of ~1.5nM for 30 min at 37°C in a humidified 5% CO_2_ incubator. A 20μl aliquot of radiotracer-plasma mixture was then added to 2 million MT4 cells in 180μl (incubation concentration ~0.15nM). After 90 min incubation on a rocker at 4°C, the total incubated radioactivity counts per minute (cpm) were measured in gamma counter (PerkinElmer 1480 Wizard 3”), microcentrifuged at 12,000 rpm for 5 min (Eppendorf 5415C), supernatant aspirated and discarded, and the cpm in the cell pellet was measured. Samples for this assay were run in duplicates.

#### Enzyme-linked immunosorbent assay (ELISA)

The presence of immunoglobulin responses directed against the anti-CD4 in the plasmas of rhesus monkeys was also determined by ELISA with minimum dilution of 1:10 and using 96-well plates coated with F(ab΄)_2_-huOKT4A. A secondary mouse anti‐human lambda light chain conjugated to biotin (Mylteni) and streptavidin/HRP system was used to detect binding of monkey IgG responses to the immobilized anti-CD4. This secondary antibody cross-reacted with rhesus lambda light chain but not to immobilized anti-CD4 which contained kappa light chains. ELISA plates were washed to remove unbound detection antibodies, followed by incubation with tetramethylbenzidine (TMB) as a substrate. Enzymatic products were measured by ELISA plate reader at 450 nm. Positive detection is defined as optical density (OD) that is at least twice the baseline value (pre-radiotracer injection).

To demonstrate the specificity and selectivity of the ATA to the radiotracer, a positive and negative control plasma were run along with the plasma samples. In addition, samples of bovine serum or 5% human serum albumin or radiotracer alone were run along with the assay samples. To test the variability and reproducibility of the assay, some plasma samples were assayed multiple times during separate assay runs with no change in the assay outcome; for such samples, the data from latest assay run was analyzed. In addition, to confirm cross-reactivity between humanized and rhesus recombinant anti-CD4 antibodies, some plasma samples were tested using both antibodies; the assay outcome was same. For quality control, a gel-filtration HPLC standard mixture containing 5 standard components with different molecular weights was also run through HPLC on the same day.

### Quantification, assay cutoffs, classification of ATA-negative and positive samples

For radio-HPLC assay, F(ab΄)_2_ (HPLC-Fab’2%) and high molecular (HM) weight immune complex (> = 250kDa; HPLC-HM%) were quantified by percent of total area under the radio-chromatogram corresponding to retention time of F(ab΄)_2_ and HM, respectively. For plasma binding assay, the percent of total incubated radioactivity bound to MT4 cells (PBA%) was determined. To correct for day-to-day variation of PBA%, HPLC-Fab’2%, HPLC-HM% in negative control, the ratio of sample to negative control was calculated (PBA-ratio, HPLC-Fab’2-ratio, HPLC-HM-ratio). Liver SUV_max_ was calculated as index of maximum radioactivity uptake in liver adjusted for body weight and injected dose. Plasma SUV was derived by multiplying the body weight with % of injected dose per mL of plasma.

Baseline plasma samples (pre-first exposure) from 36 animals were assayed to check for any pre-existing antibodies and compared to negative control. Using 95% confidence interval of baseline PBA-ratio, a sample with >0.9 PBA-ratio was defined as negative sample (no immune response). Similarly, HPLC-Fab’2-ratio >0.8 and HPLC-HM-ratio <1.4 was defined as negative sample. Based on the magnitude, antibody responses were classified into low or high. For high antibody responses, ATA-positive sample was defined as PBA-ratio <0.4, HPLC-Fab’2-ratio <0.3 or HPLC-HM-ratio >2.2. PBA-ratio between 0.45–0.65, or HPLC-Fab’2-ratio between 0.35–0.65 was defined as ATA-positive sample for low antibody responses. On occasions when negative control was not available to run along with the samples, the cut-offs were also defined using the absolute percentage—PBA% ≥45%, HPLC-Fab’2% ≥40% or HPLC-HM% ≤50% as ATA-negative sample; PBA% ≤25%, HPLC-Fab’2% ≤20% or HPLC-HM% ≥80% as ATA-positive sample for high responses. A sample is classified ATA-negative if it satisfies the cutoffs for both PBA and HPLC assays (either with ratio or absolute %) but satisfying the cutoffs for at least one of the PBA or HPLC assay (either with ratio or absolute %) was sufficient to classify the sample as ATA-positive.

### Characterization of anti-tracer antibodies

ATA-positive signal from radio-HPLC assay indicates the presence of binding antibodies. An ATA-positive signal from associated plasma binding assay indicates the presence of neutralizing antibodies, whereas an ATA-negative signal indicates the antibodies were non-neutralizing.

### Sample sizes for immunogenicity assays

A total of 393 samples were assayed with either plasma binding assay or radio-HPLC assay from all 49 animals which received F(ab΄)_2_ radiotracers. Both plasma binding assay and radio-HPLC assay were performed on 372 of 393 samples. Of these 372 samples, 341 samples were assayed with F(ab΄)_2_-CD4R1 antibody, and the remaining 31 samples assayed with F(ab΄)_2_-huOKT4A antibody. For 16 of 341 samples assayed with F(ab΄)_2_-CD4R1 antibody, negative control was not available. Hence both PBA-ratio and HPLC-ratio was available for 325 of 341 samples assayed with F(ab΄)_2_-CD4R1 antibody. Similarly, for 5 of 31 samples assayed with F(ab΄)_2_-huOKT4A antibody, negative control was not available. Hence both PBA-ratio and HPLC-ratio was available for 26 of 31 samples assayed with F(ab΄)_2_-huOKT4A antibody. In addition, 33 samples from 10 animals imaged with F(ab΄)_2_-huOKT4A were also assayed using ELISA. For these 33 samples, plasma binding assay data is available for 30 samples (from 10 animals) and radio-HPLC data for 21 samples (from 9 animals).

### Statistical analysis

The association between immune status and development of immunogenic responses over all the exposures was investigated using mixed effects logistic regression including random effects for animal, and time-varying fixed effects for SIV and TBI status, current exposure number, and the time between current exposure and 1^st^ viable immune measurement. This model provides valid inferences for longitudinal data. Samples taken less than 19 days after an exposure were not considered viable for immune measurement regardless of outcome. Only exposures occurring prior to primary response for a given animal were included in the model with the outcome (or dependent variable) being the development of an immune response (1) or not (0) for a given exposure. The association between peripheral blood cell counts or plasma viral load and development of immunogenic responses was analyzed with a similar mixed effects model, but including a time-varying fixed effect for the natural log of immune measures at exposure with and without inclusion of immune status.

All correlations are Spearman rank correlations with either bootstrap based confidence intervals and p-values for longitudinal data, or T-distribution based confidence intervals for cross-sectional data. Non-parametric Wilcoxon rank-sum test was used to compare the antibody tracer dose or plasma sampling timing between the exposures that resulted in low vs. high primary response (unpaired samples), whereas between the exposures that did or did not resulted in an immune response, robust SE was used to account for repeated measurements. All inferences were performed using R[16], and R packages lme4[17] and lmerTest[18]. P-values <0.05 were considered statistically significant. Half-life of neutralizing ATA was estimated by fitting an exponential decay curve to PBA-ratio data.

## Results

### Characterization of anti-tracer antibodies (ATA) and robustness of immunogenicity assays

Antibodies can be elicited against the variable region of other antibody molecules. Such anti-antibody responses directed against the idiotypic determinants of the injected monoclonal antibody (mAb), called anti-idiotype antibodies, are polyclonal and can either be neutralizing antibodies that binds to the active site of the infused antibody and thereby inhibit its function, or non-neutralizing antibodies that binds to the framework areas of the variable region of the infused antibody but does not affect its receptor binding activity. Since the radiotracer is a mAb, to detect the presence, specificity and characterization of immunoglobulin G (IgG) anti-tracer antibodies (ATA), a binding antibody assay (radio-HPLC) and a neutralizing antibody assay (plasma binding assay, PBA) were developed, and designed to corroborate and complement each other. In vitro, when baseline plasma was incubated with radiotracer (MW ~100kDa) and run through HPLC, high molecular (HM) weight (> = 250kDa) immune complexes were detected. Assays with bovine or human serum albumin confirmed that majority of HM in baseline sample (90–100%) was due to non-specific binding of endogenous proteins such as albumin to the radiotracer, and was not interfering with antigen binding site. When plasma containing ATA (MW ~150kDa) was incubated with radiotracer, small complexes (~250kDa) or varying sizes of immune complex chains composed of ATA and tracer (>250kDa) were formed. An increase in HM compared to baseline levels indicates the presence of binding ATA in HPLC assay. If the associated PBA also indicates ATA-positive, the ATA contain neutralizing antibodies (NATA). Alternatively, if the PBA indicates ATA-negative, the ATA are non-neutralizing (NNATA).

**Fig 1:**
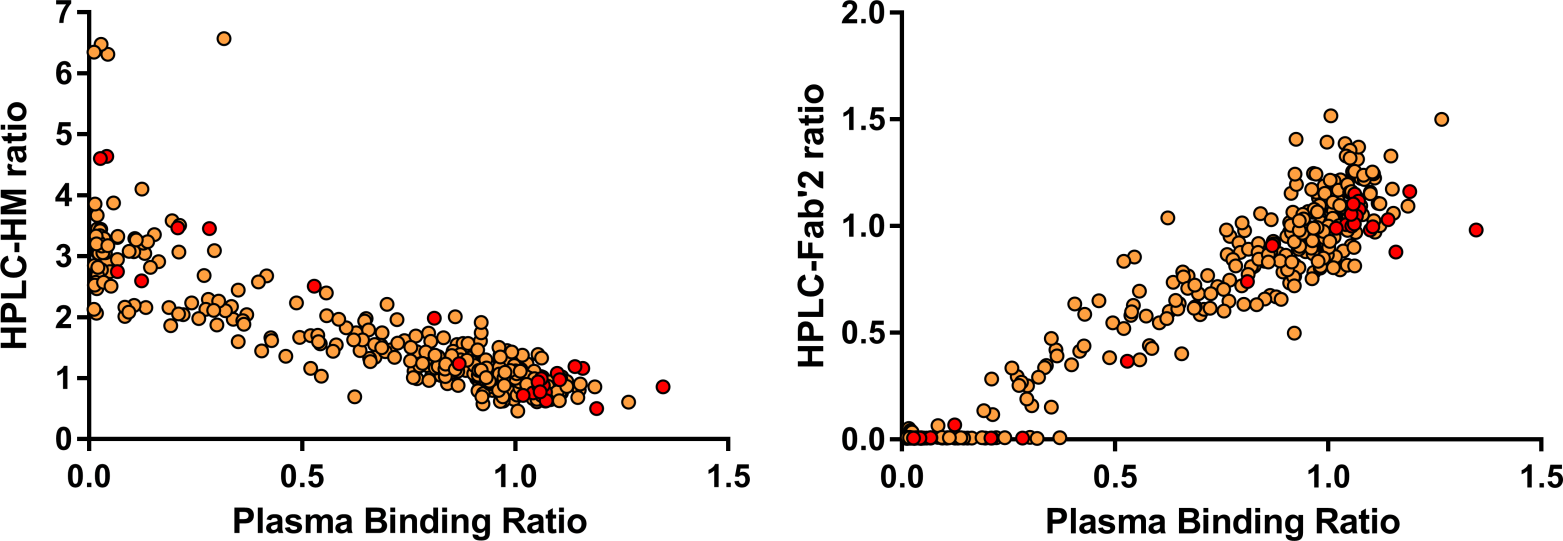
Correlation between radio-HPLC and plasma binding assay. Strong correlation was observed between plasma binding assay and HPLC assay. Samples were assayed with either F(ab΄)_2_-CD4R1 antibody (brown dots) or F(ab΄)_2_-huOKT4A antibody (red dots). For samples assayed with F(ab΄)_2_-CD4R1, the bootstrap estimate and 95% confidence interval of the Spearman rank correlation between PBA-ratio and HPLC-HM-ratio was -0.88 (-0.91, -0.84), and between PBA-ratio and HPLC-Fab’2-ratio was 0.90 (0.85, 0.93). Similar results were observed for samples assayed with F(ab΄)_2_-huOKT4A.

A total of 393 plasma samples from all 49 animals of this study were tested with PBA and/or HPLC assay in 58 assay runs over 5 years (372 samples were tested with both PBA and HPLC assays). Samples from one animal suggested the formation of only NNATA (positive signal from HPLC assay, but negative signal from PBA assay). Excluding this animal, Fig 1 shows the robustness and concurrence between the two assays irrespective of the health status of the plasma sample. This agreement between the two independent assays remained strong within each health group (data not shown), and overall for all samples assayed using F(ab΄)_2_-CD4R1 antibody, the bootstrap estimate and 95% confidence interval of the Spearman rank correlation between PBA-ratio and HPLC-HM-ratio was -0.88 (-0.91, -0.84), and between PBA-ratio and HPLC-Fab’2-ratio was 0.90 (0.85, 0.93). Similar results were observed for samples assayed with F(ab΄)_2_-huOKT4A (data not shown). A subset of these samples tested with PBA (30 samples from 10 animals) or HPLC assay (21 samples from 9 animals) were also tested using ELISA. Using PBA or HPLC assay as reference, ELISA had 100% specificity (true negative fraction) but low sensitivity (true positive fraction) to detect immune response, especially when PBA or HPLC assay showed a low response. Hence, samples were classified as ATA-positive or negative based on PBA and HPLC assays.

### Plasma sampling, tracer antibody dose and animal age

To test if an ATA response has developed, plasma samples were collected at a median of 31 days after an exposure (Interquartile range (IQR): 28–46 days). Of the 49 animals, 37 animals received F(ab΄)_2_-CD4R1, 9 animals received F(ab΄)_2_-huOKT4A, and 3 animals received both F(ab΄)_2_ antibodies for a total of 118 exposures. 35 of these exposures resulted in primary responses, 76 exposures resulted in no immune response, and the remaining 7 exposures resulted in inducing secondary response in 7 of the 35 animals that had primary response. By the end of the study, 35 macaques developed a response, and remaining 14 macaques had no detectable immune responses. Based on the magnitude, NATA responses were classified into low or high. Of the 35 primary responses, one macaque had developed NNATA, 6 had low NATA, and 28 had high NATA responses. There was no statistically significant difference in plasma sampling times for exposures resulted in primary responses (low or high NATA or NNATA; n = 35) versus those resulted in no immune responses (n = 76) (P-value = 0.8), or between exposures resulted in low (n = 6) versus high (n = 28) primary NATA responses. To check for any delayed responses, for 29 of the 76 exposures that resulted in no immune response for their first sample, a second sample was collected at a median of 28 days (IQR: 21–34 days) after the first sample was collected and tested; there were no changes in the assay outcome.

The median tracer antibody amount injected in total 118 exposures (90 exposures with F(ab΄)_2_-CDR41 and 28 with F(ab΄)_2_-huOKT4A) was 95 μg (IQR: 86–105μg). There was no statistically significant difference in the immunogenicity of developing neutralizing ATA against humanized versus rhesus recombinant antibody F(ab΄)_2_ tracers (P-value = 0.44). Also there was no statistically significant difference in the antibody amount given in exposures that led to primary response versus exposures that did not generate an immune response (P-value = 0.48), or between exposures that led to low versus high primary NATA responses. Age at 1^st^ exposure in 32 healthy controls ranged from 2.7 years to 18 years, but no statistically significant difference in the age (or gender) was observed between the 18 macaques that did, and 14 macaques that did not develop immune responses after the 1^st^ exposure. Similarly, age at 1^st^ exposure in the 17 SIV-infected animals ranged from 5 to 11.1 years, but there was no statistically significant difference in age between non-progressors (n = 5) and progressors (n = 12), or between those that did (n = 10) or did not (n = 7) develop an immune response during the study.

### Immunogenic responses in TBI, SIV-progressors or non-progressors as compared to healthy uninfected controls

After the 1^st^ exposure, 18 of 32 (56%, 95% CI: 38–73%) healthy macaques developed an immune response (15 high NATA, 2 low NATA, and 1 NNATA). Of the remaining 14 healthy macaques that did not develop a response to 1^st^ exposure, 7 underwent TBI, and 2 macaques were exposed again. Both macaques developed high NATA responses after their 2^nd^ exposure (Table 1). Among the 5 SIV non-progressors, 3 macaques (60%, 95% CI: 17–93%) developed NATA responses after the 1^st^ exposure (2 high and 1 low). Of the remaining 2 non-progressors, one macaque underwent 2^nd^ exposure and developed high NATA response. None of 12 (0%, 95% CI: 0–30%) SIV-progressors developed an immune response after the 1^st^ exposure. With 3 macaques (CD4+ T cells: 7, 84, and 199/μL) exposed only once, the other 9 progressors underwent multiple exposures (outlined in “animal study design”) as part of longitudinal studies following initiation or interruption of therapy. 6 of 9 progressors of longitudinal study developed high NATA responses after 2–5 exposures; remaining 3 macaques had no response even after 4–8 exposures. Though one animal was euthanized soon after post-transplant imaging, the remaining 6 TBI animals underwent multiple exposures during their immune recovery (outlined in “animal study design”). 5 of 6 TBI animals of longitudinal study developed immunogenic responses after ≥ 4 exposures post-irradiation; 1 animal had no response even after 5 exposures post-irradiation.

**Table 1:** Immune response outcome of total 118 exposures for all 49 macaques receiving F(ab΄)_2_ radiotracers.

Animal ID	Group	Exposure # 1	Exposure # 2	Exposure # 3	Exposure # 4	Exposure # 5	Exposure # 6	Exposure # 7	Exposure # 8
ZI10	Healthy	High, CD4:645; CD20: 968							
RQ7387	Healthy	High, 1028;960							
A10X002	Healthy	High, 2335;1123							
ZJ05	Healthy	High, 2065;845							
GLM	Healthy	High, 530;235							
A8E032	Healthy	High, 884;464							
DCDK	Healthy	High, 1524;863							
H572	Healthy	High, 982;282							
DCAK	Healthy	High, 1280;1153							
DCET	Healthy	High, 438;508							
DBIJ	Healthy	High, 1057;440							
ZC08	Healthy	High, 756;511							
CF67	Healthy	High, 859;243							
XAW	Healthy	High, 841;539							
CE7E	Healthy	High, 1277;313							
ZD21	Healthy	Low, 693;368							
DBPN	Healthy	Low, 704;548	Secondary, 584;414						
DCAE	Healthy	No, 1692;1087	High, 1207;718						
01D278	Healthy	No, 927;610	High, 685;437						
A9E005	Healthy	Non-neutralizing, 1626;1007							
RQ7280	Healthy	No, 1068;464							
H550	Healthy	No, 488;216							
EV3	Healthy	No, 1513;1175							
K36	Healthy	No, 1085;849							
DC4P	Healthy	No, 2031;1480							
DCBP	SIV non-progressor	High, 1076;1446	Secondary, 963;851						
A5E036	SIV non-progressor	High, 1326;446							
P707	SIV non-progressor	Low, 1718;1029	Secondary, 718;1122						
A5E006	SIV non-progressor	No, 1285;392	High, 1001;408						
P887	SIV non-progressor	No, 838;298							
P247	SIV-progressor	No, 32;348	High, 116;626						
P248	SIV-progressor	No, 69;106	No, 472;177	High, 1008;303					
P261	SIV-progressor	No, 87;536	Low, 304;651	Secondary, 321;461					
P844	SIV-progressor	No, 315;718	No, 266;554	No, 368;713	High, 548;910				
P731	SIV-progressor	No, 356;181	No, 608;311	No, 335;198	High, 489;369				
P881	SIV-progressor	No, 1488;556	No, 978;397	No, 1077;853	No, 773;593	High, 875;502			
G43	SIV-progressor	No, 7, 219							
DA24	SIV-progressor	No, 84;218							
P636	SIV-progressor	No, 199;701							
P773	SIV-progressor	No, 206;375	No, 154;314	No, 184;550	No, 442;708				
H755	SIV-progressor	No, 116;468	No, 144;391	No, 260;898	No, 374;1455	No, 307;1145	No, 339;910	No, 321;573	No, 339;542
P246	SIV-progressor	No, 296;445	No, 210;299	No, 46;427	No, 172;767	No, 321;474	No, 510;513		
ZI12	Healthy/TBI	No, 1784;930	No, 93;0	No, 584;139	No, 1279;907	High, 1190;881	Secondary, 1116;427		
ZG21	Healthy/TBI	No, 1693;735	No, 51;0	No, 212;67	No, 417;1495	Low, 1577;1062	Secondary, 1093;415		
ZH32	Healthy/TBI	No, 1028;839	No, 72;2	No, 314;328	No, 931;3229	No, 582;1910	Low, 1217;1592	Secondary, 969;1201	
ZG41	Healthy/TBI	No, 595;343	No, 20;0	No, 175;48	No, 261;160	No, 556;953	No, 1328;1199	High, 789;520	
ZG70	Healthy/TBI	No, 1269;513	No, 2;0	No,121;127	No, 458;1162	No, 603;915	No, 1001;837	High, 1115;979	
ZI64	Healthy/TBI	No, 1077;1672							
ZJ37	Healthy/TBI	No, 873;939	No, 10;0	No, 344;244	No, 573;2037	No, 442;2243	No, 685;2363		

For each exposure, immune response outcome of that exposure and peripheral blood CD4+ and CD20+ counts/μL at exposure are shown. “No” indicates no immune response (n = 76), “High” indicates high titer primary neutralizing antibody response (n = 28), “Low” indicates low titer primary neutralizing antibody response (n = 6), “Non-neutralizing” indicates non-neutralizing antibody response (n = 1), and “Secondary” indicates a secondary antibody response (n = 7).

Since for therapeutics (or vaccines), the incidence of antibody response is less relevant than the magnitude and characterization of response on efficacy (or protection) and outcome, the odds of developing high NATA responses were compared among the groups. In a mixed effects model adjusting for the number of exposures, the odds of developing immunogenic responses was siginificantly lower in TBI (odds ratio (OR) = 0.022, P-value <0.005) or SIV-progressors (OR = 0.02, P-value <0.005) as compared to healthy. The odds were similar in SIV non-progressors and healthy animals (OR ≈1). There was no significant difference in the odds between TBI and SIV-progressors (P-value = 0.9). Similar results were observed for the likelihood of producing any level/type of response (either low or high NATA, or NNATA).

### Immunocompetence vs. peripheral blood (PB) cell counts

**Fig 2:**
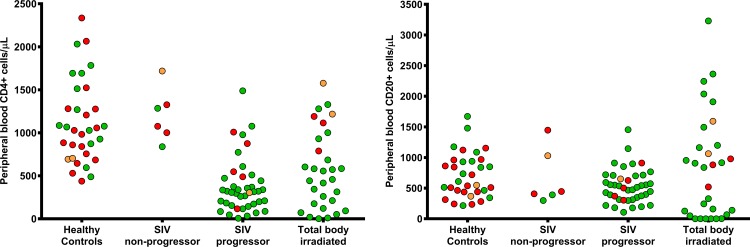
Immunocompetence vs. peripheral blood CD4+ and CD20+ cell counts. Peripheral blood (PB) CD4+ cell counts at exposure, but not CD20+ cell counts, were significantly and positively associated with odds of development of primary antibody responses (P<0.001). Plots show PB CD4+ and CD20+ cell counts at exposure, and the outcome of the exposure where green dot indicate no immune response, orange dot indicate low neutralizing primary antibody response, and red dot indicate high neutralizing primary antibody response.

In a mixed effects model over all 49 animals of this study (PB counts range at exposure, CD4+: 2 to 2335 cells/μl, CD20+: 0 to 3229 cells/μl), CD4+ counts at exposure, but not CD20+ levels, were significantly associated with odds of developing an immune response (Odds Ratio for log_e_(CD4+) = 3.24, P <0.001) with increasing CD4+ counts increasing the odds of immune response (Fig 2). Immune responses were not observed in animals with < = 100 CD4+ cells/μl at exposure. Limiting the analysis to exposures with >100 CD4+ cells/μl, we found a significant association between CD4+ count and increased odds of immune response (OR for log_e_(CD4+ >100 cells/μl) = 2.2, P-value <0.01). This significance with CD4+ count was lost when the analysis was limited to CD4+ ≥750 cells/μL or when limited to only healthy animals.

When the analysis was limited to SIV-infected animals, cART therapy status (ON or OFF at exposure) was not statistically significant (P-value = 0.26), and PB CD4+ count was a stronger predictor of response than plasma SIV-RNA levels (OR for log_e_(CD4+) = 3.63, P-value = 0.03; OR for log_e_(viral load) = 0.83, P-value = 0.2). Similar results were observed in TBI group with increasing CD4+ levels boosting the likelihood of responses, but likely due to small sample size, this observation was not statistically significant. Using a similar mixed effects model but adjusting for CD4+ counts and comparing to healthy, both SIV-progressors and TBI were still functionally incompetent in generating a response (OR for SIV progressor = 0.06, OR for TBI = 0.05, P-value <0.01), and were not significantly different from one another; whereas SIV non-progressors have similar odds to healthy controls (OR for SIV non-progressor = 1.06, P-value = 0.94). Results were similar when immune response was defined as development of any level/type of response (either low or high NATA, or NNATA).

### Clearance of anti-tracer antibodies

**Fig 3:**
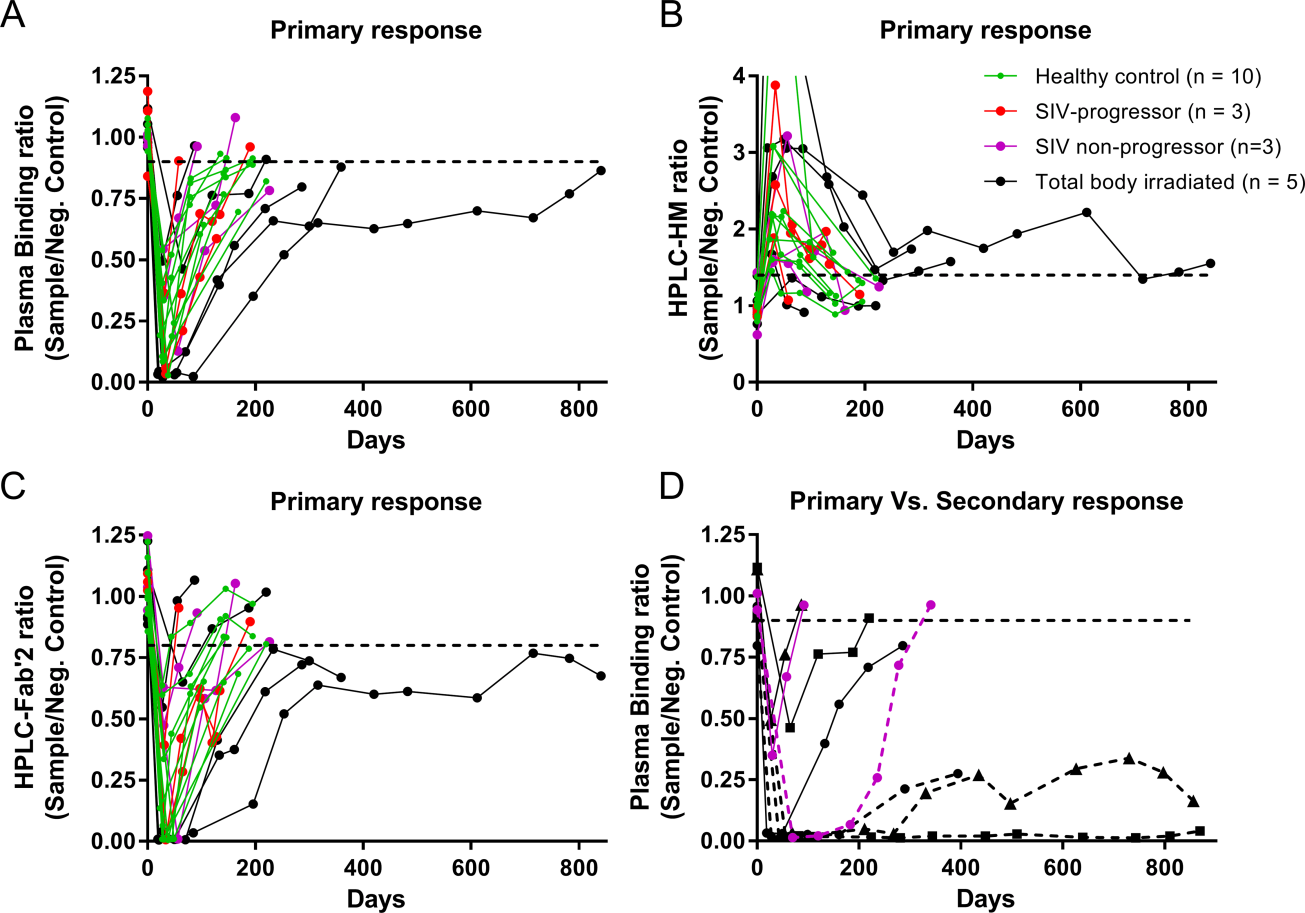
Clearance of endogeneous anti-tracer antibody (ATA). Anti-tracer antibody clearance from primary response in 10 healthy uninfected controls (green solid), 3 SIV-progressors (red solid), 3 SIV non-progressors (purple solid), and 5 total body irradiated (black solid) rhesus macaques from (A) plasma binding assay, and (B) (C) radio-HPLC immunogenicity assay using F(ab΄)_2_-CD4R1 antibody. (D) shows clearance of anti-tracer antibody from primary and secondary responses in one SIV non-progressor (purple) and 3 total body irradiated (black) rhesus macaques using plasma binding assay where solid lines indicate ATA clearance from primary response, and dashed lines indicate ATA clearance from secondary response. Day 0 indicates the radiotracer exposure.

ATA clearance from primary response was estimated in 21 of 35 macaques that developed an immune response (10 healthy controls, 3 SIV-progressors, 3 SIV non-progressors, and 5 TBI animals). Plasma samples obtained after ~4 weeks from exposure were assayed from 10 healthy macaques (2 low and 8 high NATA), and the median ATA half-life estimated was 56 days (IQR: 44–67 days; range: 32–91 days) (Fig 3A–3C). Though some SIV animals had very fast clearance, the ATA clearance in 3 SIV-progressors (half-lives 10, 61 and 76 days) and 3 non-progressors (half-lives 15, 69 and 88 days) appear to be similar to healthy controls. The ATA clearance in the 5 TBI animals was highly variable. 2 TBI macaques with low response had clearance with half-lives of 16 and 72 days; remaining 3 macaques with high responses continued to have high ATA levels (above the assay threshold) for 7–12 weeks after the exposure and then started to decline with half-lives of 101 and 129 days; the last macaque showed a 2-phase decay with half-lives 156 and 372 days. Although there is some evidence in the TBI group of a positive association between level of immune response and ATA half-life, this association did not extend to healthy or SIV-infected animals, suggesting that either TBI treated animals differ in some way, or in general ATA level and half-life are not strongly associated. The 3 TBI macaques with high responses had significantly longer ATA half-lives as compared to healthy animals (P-value <0.01). In addition, over all 21 animals for which the ATA half-life was estimated, no significant association was observed between ATA clearance and age or body weight.

After ATA were cleared from plasma, 7 animals (1 healthy, 1 SIV-progressor, 2 SIV non-progressor, and 3 TBI) that had a primary response (5 low and 2 high NATA) were exposed again to induce a secondary response (Table 1), and 4 of them (1 SIV non-progressor and 3 TBI) were followed longitudinally. As expected, the secondary responses were enhanced- antibody levels were high and the production continued for a longer period with ATA levels persisting for months or years (Fig 3D).

### Altered in-vivo biodistribution in the presence of anti-tracer antibodies

**Fig 4:**
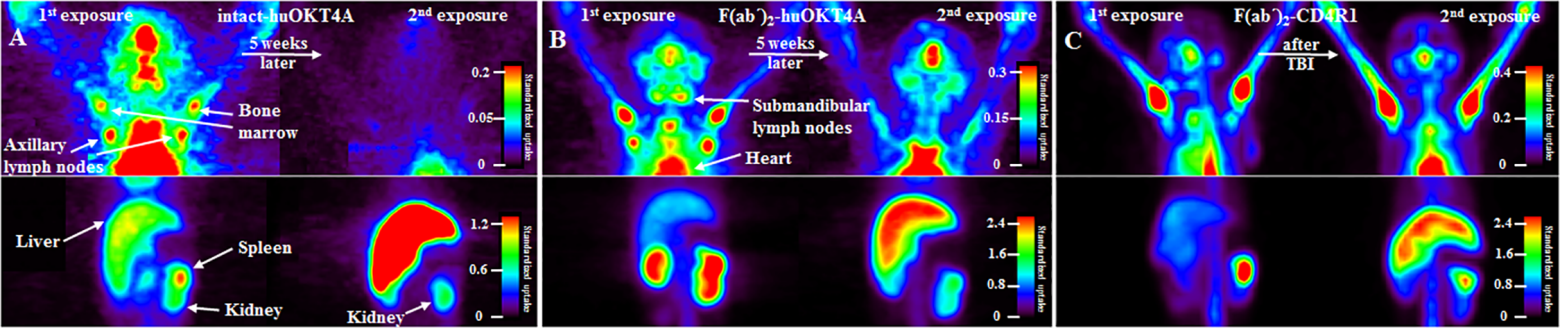
Maximum intensity projection SPECT images. Rhesus macaques that developed antibody response to the radiotracer after baseline exposure were subsequently imaged while anti-tracer antibodies were present in the plasma in (A) healthy macaque imaged at 48 hours post-radiotracer injection with intact-huOKT4A labelled with ^111^In and scanned using Triad88 (Trionix) camera, (B) SIV-TK infected non-progressor imaged at 4 hours post-radiotracer injection with F(ab΄)_2_-huOKT4A labelled with ^99m^Tc and scanned using Triad88 (Trionix) camera, and (C) healthy macaque that underwent total body irradiation prior to 2^nd^ exposure and imaged at 4 hours post-radiotracer injection with F(ab΄)_2_-CD4R1 labelled with ^99m^Tc and scanned using Symbia T2 (Siemens) camera. Images show increased hepatic uptake and altered biodistribution with minimal to no binding observed in secondary lymphoid organs when imaged in the presence of anti-tracer antibodies. Within each panel, images were adjusted (standardized) for injected dose and body weight. Tissue uptakes were converted to RAINBOW color scale as shown in color bar.

In nuclear medicine imaging, low levels of transiently expressed ATA are not of concern as they rarely impact clinical outcomes. Though both NATA and NNATA can alter the pharmacokinetics of the tracer by influencing its clearance, the development of high titer, high affinity ATA that interfere with the radiotracer are of significant concern. Few animals in our imaging program were imaged while ATA were present in plasma (high or low titers) with either intact-huOKT4A or with F(ab΄)_2_ fragments of huOKT4A or CD4R1. 3 macaques that developed high NATA responses to the first exposure with intact-huOKT4A underwent 2^nd^ exposure 5 weeks later with the same radiotracer. Presence of ATA in PB at the time of 2^nd^ exposure altered the *in-vivo* biodistribution visualized from SPECT images as rapid blood clearance (heart pool activity), high hepatic uptake, and no binding in targeted tissues (lymphoid organs for anti-CD4 radiotracers used in this study). Liver uptake (Maximum Standardized Uptake Value (SUV_max_)) at 2^nd^ exposure increased by ~4-fold as compared to 1^st^ exposure (Fig 4A).

For F(ab΄)_2_ radiotracers, presence of ATA at exposure increased the amount of radiotracer (quantified as SUV) in blood and plasma measured at 6h post-radiotracer injection, and also enhanced the plasma half-life of the tracer from ~12hours to up to 36hours. Liver SUV_max_ increased by at least 2-fold, and very low binding was observed in secondary lymphoid tissues (Fig 4B and 4C). In a mixed-effects model over all animals imaged with F(ab΄)_2_ radiotracers, presence of neutralizing ATA in high titers at imaging was positively associated with increased plasma SUV (P-value <0.001) and liver SUV_max_ (P-value = 0.047). Of note, one healthy macaque that developed high NATA response after 1^st^ exposure to F(ab΄)_2_-CD4R1 underwent TBI (3Gyx2) ~10weeks later, and was imaged again at 6 days post-TBI. Soon after TBI, ATA were still present in plasma at high enough titers and altered the biodistribution (Fig 4C).

## Discussion

Antibody-based imaging is based on its inherent ability to specifically bind to a target with high affinity, and as such the formation of neutralizing anti-tracer antibodies are detrimental to diagnostic imaging. Imaging of tissue CD4 pool is a relatively recent approach in the field of immunology and has highlighted the importance of *in vivo* imaging for inference on the state of immunocompetence of the host compared to inference derived from peripheral blood compartment[14, 15, 19]. But the ability to conduct longitudinal studies is limited due to the development of immune response against the mAb radiotracer. Eliminating antibody Fc portion is generally considered to result in less immunogenic fragments (Fab or F(ab΄)_2_), in part due to their faster systemic clearance, with F(ab΄)_2_ fragment superior to whole immunoglobulin or Fab fragment due to reduced immunogenicity and being divalent[20, 21]. Both radiotracers used in this study are CDR (of murine)-grafted mAbs. While humanized anti-CD4 (huOKT4A) is completely foreign to rhesus host, rhesus recombinant anti-CD4 (CD4R1) has some foreign content. Only 3 healthy (uninfected controls) macaques were exposed with intact-huOKT4A, and all 3 macaques developed high neutralizing ATA responses to first exposure. To reduce the risk of immunogenicity, we transitioned to F(ab΄)_2_-huOKT4A, and eventually to F(ab΄)_2_-CD4R1. In this study, the incidence (likelihood) of immunogenic responses, and levels (titer) of anti-tracer antibodies were similar between animals exposed with humanized versus rhesus recombinant F(ab΄)_2_ fragments. Hence, primatization of humanized anti-CD4 did not appear to be less immunogenic in monkeys, but only resulted in an improved biodistribution. Immunogenic responses are species dependent and animal models are not completely predictive of immunogenicity to a given protein in humans[22]. This study was designed not as a tool to predict relative immunogenicity across species, but to evaluate the potential and determine the host factors of induction of immune responses by foreign protein antigen in immunocompetent and immunocompromised groups within species.

Compared to PBA and HPLC assays, ELISA, the most commonly used immunoassay, appears to be less sensitive. Here, we found no evidence of number of prior exposures being significantly associated with increased risk of immune response to subsequent exposure. In other words, odds of induction of immunogenic responses were similar between the animals that were not previously exposed, versus, animals previously exposed but with no detectable immune responses, indicating that PBA and HPLC assays are sensitive enough to not miss any low immune responses.

In adults with mature immune systems, induction of immune responses are influenced by many characteristics of the antigen, administration, and host factors. The more foreign the antigen, the more immunogenic it is. While higher dose and multiple administrations (but allowed to clear before subsequent administration) are required to stimulate a strong response, continuous exposure to high levels of circulating antigen may induce tolerance[23, 24]. For a given antigen, the route of entry can affect the outcome as this determines not only which organs and cell populations are involved in the response, but also the elimination half-life of antigen. Host factors involved depend on the activation pathway. Exogenous protein antigens trigger CD4+ T-cell responses, and the presence of affinity matured, class switched IgG isotype, anti-idiotype, anti-tracer antibodies indicates that the radiotracer is driving a T-cell dependent (TD) mode of immune response. For TD antigens, B-cell activation involves stimulation of multiple components of immune system and complex interplay among antigen presenting cells (APC), T-helper cells, secreted cytokines, co-stimulator molecules and B-cells. Finally, other host related determinants are age and gender. T-helper cells activated during the primary immune response are naïve, and thymic involution with aging diminishes thymus size leading to decline in naïve T-cell output. In this study, there was no significant difference in the age (or gender) within the healthy macaques that did or did not developed immune responses after the 1^st^ exposure. Similarly, there was no significant difference in age between SIV-progressors and non-progressors, or between those that did or did not develop an immune response during the study. Besides administration route being the same (intravenous), and antigen (radiotracer) dose similar for all subjects and for all exposures, animals were exposed within a 3 hour window in the afternoon, thus, excluding the potential time-of-day dependence of adaptive immune responses[25, 26]. Moreover, antigen processing/presentation and MHC expression in HIV-infected patients was shown to be not impaired, and defective only in final stages of disease[27]. Hence, any differences in the ability to generate antibody responses in the groups must be host related T-cell/B-cell defects and repertoire.

We recently re-confirmed that peripheral blood (PB) is a tiny window into total body lymphocytes/subsets, and secondary lymphoid tissues, where host immune responses occur, are not accurately mirrored by PB counts when perturbed from quasi-steady state of the immune system[14, 15]. Nevertheless, for ease of access and sampling, PB counts are widely used as surrogate markers even for diseases of lymphoid tissues like HIV/SIV. The number of B-cells, the progenitors of plasma cells, should be correlated with humoral responses for both TD and TI antigens. In this study, PB CD4+ cell count alone had strong positive association with the development of immune responses, and PB CD20+ cell count remained non-significant even after adjustment for CD4+ count. This could be because for TD antigens, B-cell activation and differentiation require T-cell help, or B-cell and T-cell counts are highly associated longitudinally in the animals recovering from TBI; although we did not find an association in SIV-progressors. Since immune responses were not observed when PB CD4+ counts <100/μl, and significant association with development of immune responses was lost when PB CD4+ counts >750/μl, we analyzed the odds ratio for CD4+ counts between 100-750/μl. An *e*-fold (2.718) increase of PB CD4+ counts is associated with an odds ratio of 2.2. This translates to an odds ratio of 1.73 for doubling the PB CD4+ counts. Interestingly, in concurrence with the above observation, whole-body CD4 imaging showed non-significant differences in the CD4 pool of secondary lymphoid organs when PB CD4+ counts were >750/μl.

During HIV/SIV infection, lymphoid tissue architecture is largely destroyed, and along with chronic immune activation and B-cell dysfunction, the complex interaction required for humoral response is disrupted[28]. Both, T- and B-cell abnormalities, and loss of CD4+ T-cells associated with high plasma viremia, contribute to poor antibody responses to TD immunizations[29]. Before the era of antiretrovial therapy (ART), historical TD vaccination studies in HIV patients showed complete lack of, or extremely impaired antibody responses in patients with severe immunodeficiency (CD4+ T-cells <300/μL)[30–32], while protective levels of antibody responses were observed in long-term non-progressors[28, 33]. Since non-progressors maintain immunity (high CD4+ T-cell counts) even in the absence of antiretroviral treatment as compared to progressive loss of CD4+ T-cells in progressors, SIV-infected animals were divided into progressor and non-progressor groups in this study. Though ART can restore CD4+ T-cell counts and reduce plasma viremia, the functional immune defects persist and do not fully revert with therapy, leading to improved but weak responses even in the context of total virologic suppression[27, 34]. When the analysis was limited to SIV-progressors in our study, increasing PB CD4+ count or decreasing plasma viral load after cART initiation was associated with increased odds of immune response, indicating the mechanism of TD responses seem to be intact and appear to recover to a functional level. However, compared to healthy animals with similar CD4+ counts and number of exposures, SIV-progressors were still associated with lower odds of generating a response. Moreover, in the mixed effects model for SIV group (including or excluding non-progressors in the model) and adjusting on CD4+ counts at exposure, being ON therapy at exposure did not seem to have a significant influence on the odds of generating an immune response as compared to being OFF therapy at exposure, thus justifying combining the animals into SIV-progressor or non-progressor groups irrespective of the therapy status.

Total body irradiation induces bone marrow ablation. Though the immune system is eventually reconstituted by hematopoietic stem cell transplanation, the recovery is heterogenous and sub-optimal among lymphoid tissues as compared to peripheral blood compartment[14]. When the analysis was limited to TBI treated animals, increasing PB CD4+ levels boosted the likelihood of responses, indicating that the functionality of the immune system arising from stem cell transplantation seem to be unharmed. But for similar PB CD4+ counts and number of exposures, TBI treated animals were still associated with lower odds of generating an immune response as compared to healthy animals.

In humans, therapeutic (except murine mAbs) and endogenous IgG have long elimination half-lives (up to 4 weeks) due to antibody recycling by neonatal Fc receptor[9, 35]. Similary, in rhesus monkeys, intact IgG is protected from degradation (half-life ~8 days)[36]. In contrast, radiotracers in this study are F(ab΄)_2_ fragments (no Fc portion), and is eliminated relatively rapidly in the plasma (half-life ~12 hrs). Intravenous (IV) route of antigen administration is generally considered to be least likely to induce systemic level immune response. IV administered TD antigens can either induce immune unresponsiveness (tolerance), or, if presented by activated APC with enough co-stimulation, an immune response is generated in the spleen. The physiology of primary TD antibody responses in healthy subjects is well defined[37, 38]- usually within a day of antigen entry, due to crosslinking of antigen-specific naïve B-cell receptors by antigen displayed by follicular dendritic cells (FDC) or soluble antigen, B-cell starts to migrate to the edge of follicle towards the T-cell area. Simultaneously, antigen-specific naïve T-helper cell (T_H_), in response to interactions with resident mature dendritic cells of the spleen presenting the processed antigen through peptide-MHC class II complexes, and co-stimulatory signals from the same APC, is activated, and undergoes rapid clonal expansion[39]. During this proliferation, some primed T_H_ cells migrate towards the follicle and fully activate antigen-specific B-cells. 1–2 days following this cognate B- and T-cell interaction, some activated B-cells differentiate into plasma cells. Thus, after an initial lag of 4–6 days from antigen exposure, short-lived, low affinity IgM antibodies are secreted at these extrafollicular sites. Alternatively, other activated B-cells re-enter the follicle along with cognate T-follicular helper cells (T_FH_) and a germinal center (GC) develops. In GC, activated B-cells proliferate rapidly and as a result of interactions with FDC, co-stimulator pairs and the cytokine environment produced by T_FH_ cells, B-cells undergo somatic hypermutation and class switch recombination[40]. After a second lag phase of 4–5 days from the start of GC, B-cells differentiate to plasma cells (short-lived and long-lived) or memory B-cells. The short-lived plasma cells secrete huge amounts of high affinity IgG antibodies but has a life-span of ~5 days, whereas, long-lived plasma cells (LLPC) migrate to bone marrow and continue to produce modest amounts of antibodies. The GC reaction disappears in ~3 weeks. Hence, during primary TD responses in an healthy animal injected with a relatively fast clearing antigen (half-life ~12 hrs), the production of ATA by short-lived plasma cells ceases by ~4 weeks from exposure. Given the half-life of endogenous IgG in rhesus monkeys of ~8 days[36], it seems plausible that ATA levels 4 weeks after the exposure were maintained by LLPC and the measured decline of median half-life ~56 days in healthy macaques also roughly corresponds to the decay of LLPC. In 5–7 months from exposure, ATA from primary response could not be detected in plasma of healthy animals. It should be noted that absence of circulating ATA does not imply absence of immunity. Immunological memory, in which both class-switched memory B-cells and memory T-cells generated during primary TD response persist for years or life-long which can be easily activated to provide rapid and enhanced responses during subsequent exposures to the same antigen.

The 3 SIV-progressors and 3 non-progressors followed during the primary ATA clearance showed similar ATA half-lives as of healthy controls, and had PB CD4+ count >480/μL and >1000/μL, respectively at exposure. ATA half-life estimates in CD4+ depleted (<300/μL) SIV animals were not available in this study. It appears that the TBI animals with high responses had significantly longer ATA half-lives as compared to healthy, and thus likely longer survival of LLPC. In addition, one healthy macaque that developed high NATA response to its 1^st^ exposure and subsequently underwent TBI (3Gy x 2), ionizing radiation showed no affect on ATA levels in systemic circulation which cleared similar to ATA decay rate in healthy animals, corroborating earlier studies that plasma cells are radioresistant[41].

Immunogenicity assays indicate that anti-tracer antibodies in the plasma immediately form high molecular weight immune complexes with the injected radiotracer. Our *in vivo* CD4 imaging in the presence of ATA is consistent with tumor studies in patients which showed that these immune complexes are cleared rapidly by the liver resulting in high hepatic uptake, altered blood clearance, and reduced target uptake of the radiotracer[42, 43]. For intact-huOKT4A mAb radiotracer (plasma half-life ~5 days), immune complexes resulted in rapid blood clearance, ~4-fold increase in hepatic uptake, and no uptake in lymphoid tissues. In contrast, for Fab’2 radiotracers (plasma half-life ~12 hours), immune complexes slowed blood clearance of the tracer, hepatic uptake increased by ~2-fold, and minimal uptake was noted in lymphoid tissues. The observed difference in the liver uptake increase between intact and Fab’2 mAb tracers is likely related to the ATA titers developed and the size of the immune complexes formed with the mAb tracer used, but is independent of the health status of the animal.

In summary, tracer doses of therapeutic antibodies (~3 log lower than therapeutic levels) administered intravenously can induce immune responses. Since ATA in systemic circulation have the potential to completely neutralize the tracer and prevent the binding to the target site, there is a need to measure (using high sensitivity assays) and assess immunogenic responses to antibody tracers from prior exposures, and even at baseline for any pre-existing or cross-reactive anti-tracer antibodies. SIV-infection and total-body irradiation is associated with more profound T-cell (and possibly B-cell) dysfunction which seems to persist and does not fully revert even with successful recovery of peripheral blood counts. Compared to healthy controls or SIV non-progressors with similar CD4+ count, both SIV-progressors and TBI macaques seemed to be still functionally incompetent in generating an immune response.
